# Somatic mutations during rapid clonal domestication of *Populus alba* var. *pyramidalis*

**DOI:** 10.1111/eva.13486

**Published:** 2022-10-04

**Authors:** Zeyu Zheng, Hongyin Hu, Weixiao Lei, Jin Zhang, Mingjia Zhu, Ying Li, Xu Zhang, Jianchao Ma, Dongshi Wan, Tao Ma, Guangpeng Ren, Dafu Ru

**Affiliations:** ^1^ State Key Laboratory of Grassland Agro‐Ecosystems, College of Ecology Lanzhou University Lanzhou China; ^2^ Key Laboratory of Bio‐Resource and Eco‐Environment of Ministry of Education, College of Life Sciences, State Key Laboratory of Hydraulics and Mountain River Engineering Sichuan University Chengdu China

**Keywords:** clonal, deleterious, heterozygous, *Populus alba* var. *pyramidalis*, somatic mutation

## Abstract

For many clonally propagated species, the accumulation of somatic mutations is the principal driver of declines in yield and quality. However, somatic mutations may also promote genetic diversification. Thus, elucidating somatic mutation rates and patterns is important to understand the genetic basis undergirding the emergence of commercially valuable traits and developmental processes. In this study, we studied the effect of short‐time clonal domestication of *Populus alba* var. *pyramidalis*, a species that has been propagated by cutting for the last 67 years. We found that: (1) the somatic mutation rate for *P. alba* var. *pyramidalis* is 9.24 × 10^−9^, which is higher than rates observed in related species; (2) there were more mutations near heterozygous regions, and a larger proportion of CpG and CHG sites were associated with somatic mutations, which may be related to the blocking of DNA repair by methylation; and (3) deleterious mutations were not shared by multiple individuals, and all occurred in heterozygous states, demonstrating the strong selective pressures that act against deleterious mutations. Taken together, the results of our study provide a global view of somatic mutation that will aid efforts to understand the genetic basis of commercially valuable traits and to improve clonally breeding species.

## 1 INTRODUCTION

The pattern and impact of somatic mutations has been one of the most intensely debated subjects in evolutionary biology for the past 30 years (Gill et al., 1995; Walbot, 1985; Whitham & Slobodchikoff, 1981). Some have argued that somatic mutations, as a major source of genetic diversity in clonal evolution, are beneficial drivers of innovation for plant evolution (Gaut et al., 2015; McKey et al., 2010; Miller & Gross, 2011). For example, in artificial breeding programs, somatic mutations often provide novel genetic material for the creation of bud sport mutants among clonal offspring (Benedict, 1923; Foster & Aranzana, 2018; Shamel & Pomeroy, 1936). Recent work has also found that somatic mutations have played an important role in reducing the fruit acidity among orange cultivars (Wang et al., 2021). Moreover, somatic mutations appear to have been important to the evolution of the well‐known nectarine variant of peach (Wen et al., 1995) as well as the origin of modern grape cultivars (Laucou et al., 2011). On the other hand, some studies in evolutionary biology have concluded that somatic mutations are mainly deleterious, that the accumulation of such mutations is a fundamental aspect of plant senescence, and that somatic mutation interferes with breeding for agricultural purposes. For example, cannabis producers have noted lower‐quality clones taken from a mother plant that often result in reduced cannabinoid production and plant vigor (Adamek et al., 2021). Furthermore, studies in potato (Zhang et al., 2019), cassava (Ramu et al., 2017) and banana (Li & Ge, 2017) have noted a significant accumulation of recessive deleterious mutations during long‐term clonal reproduction, leading to more than 60% decrease in root yield after inbreeding. Both new evidence and observations of well‐known phenomena suggest that plant vigor decreases during extended periods of asexual reproduction (Muller, 1932).

By some estimates, approximately two‐thirds of temperate plant species are capable of clonal propagation in nature (De Kroon & Van Groenendael, 1990; Klimeš et al., 1997; Schoen & Schultz, 2019). In theory, clone‐produced plants are genetically identical to and phenotypically similar to the parent stock. In reality, however, clonal reproduction does not result in perfect replication, in part due to the accumulation of somatic mutations. For example, woody species propagating vegetatively in nature, such as *Robinia pseudoacacia* (Lian et al., 2004), *Populus tremuloides* (Ally et al., 2008), *Populus nigra* (Chenault et al., 2011) and *Populus trichocarpa* (Hofmeister et al., 2020), or species propagating in ex situ conditions, such as *Olea* spp. (Lopes et al., 2009), *Picea abies* (Helmersson et al., 2008), *Vitis vinifera* (Crespan, 2004; Vezzulli et al., 2012) and *Pinus pinaster* (Marum et al., 2009), are often accompanied by somatic mutations. The domestication of agricultural plants (e.g., grape, hop, banana, potato and coffee) has also relied heavily on clonal reproduction (Carrier et al., 2012; McKey et al., 2010). However, the pattern and impact of somatic mutations such as mutation rate, distribution, morphological effects and changes in vigor in cloned perennial plants remain poorly understood (Schoen & Schultz, 2019). One reason is the inherent difficulty in detecting somatic mutations. Recent advances in genome sequencing technologies and functional genomics (Alonso & Stepanova, 2016) now make it possible to increase the detection rate of somatic mutations; still, only a few studies have used genomic approaches to investigate somatic mutations in clonal plants (Carbonell‐Bejerano et al., 2017; Carrier et al., 2012; Gambino et al., 2017; Plomion et al., 2018; Roach et al., 2018).

When a clonal offspring is produced, somatic mutations will inevitably accumulate and may be unequally distributed across the plant's genome (Yu et al., 2020). Understanding the extent to which somatic mutation rates and patterns occur across the tree of life may help to clarify their role in shaping organismal longevity and/or in shaping the adaptive potential of populations. Here, we focused on the incidence rate and pattern of somatic mutations found in colonies of *Populus alba* var. *pyramidalis*, which was first described in the Karatao Mountains of Kazakhstan in 1854 (Fang et al., 1999; Nauk, 1854). In addition to the plant's straighter, smoother and whiter trunk, the smaller angle occurring between trunk and branches makes this variety easily distinguishable from *P. alba*.

Since its discovery in Xinjiang province, China, last century by Chinese scientists, *P. alba* var. *pyramidalis* has been widely cultivated for urban afforestation and ecological restoration in North China by branch cuttings (Xu, 1988; Yin, 2006; Zhang et al., 2008). In 1981, it was reported that more than 1.75 million clones had been propagated within a 8.8 km^2^ man‐made forest (Cai et al., 1983). Even more noteworthy, there are no female individuals observed in *P. alba* var. *pyramidalis*, which prevents the species from generating catkins in summer. Some studies have shown that catkins can carry up to 6.33 × 10^4^ bacterial and 7.46 × 10^5^ fungal cells (Xu & Yao, 2020) and cause allergic reactions and respiratory disease (Buters et al., 2008; Katz et al., 2020; Kim, Kwak, et al., 2020). In addition, whirling catkins can reduce visibility, affecting the accuracy of some high‐precision instruments (Tang et al., 2014). Thus, *P. alba* var. *pyramidalis* is an excellent alternative tree species. The genome of *P. alba* var. *pyramidalis* has been published (Ma et al., 2019), and it has been well‐studied in the context of alternative spicing (AS) events (Hu et al., 2020) and salt tolerance (Chen et al., 2020). However, as exclusively clonally propagated long‐lived perennials, the rate and pattern of somatic mutation, which have great effect on population‐level variation in an eco‐evolutionary context, are largely unknown. In this study, we use whole‐genome resequencing data for 11 *P. alba* var. *pyramidalis* and 28 *P. alba* individuals to answer the following questions: (1) does clonal propagation increase the rate of somatic mutation in *P. alba* var. *pyramidalis* relative to related species; (2) which genomic regions are more prone to somatic mutations; and (3) what is the pattern of deleterious somatic mutations?

## 2 MATERIALS AND METHODS

### 2.1 Sample collection, sequencing and mapping

**FIGURE 1:**
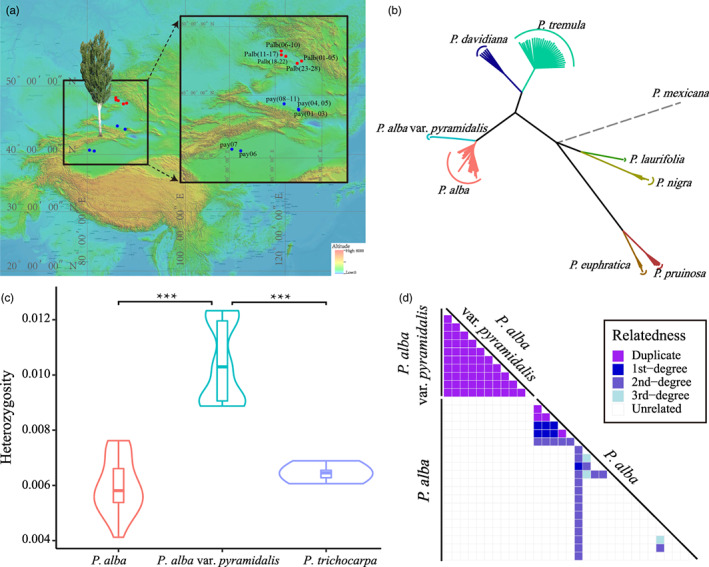
Sample location and relatedness. (a) Sampling locations for *P. alba var. pyramidalis* (pay, in blue points) and *P. alba* (Palb, in red points). (b) Phylogenetic relationship between *P. alba* var. *pyramidalis*, *P. alba* and six other species that are distributed across Xinjiang province. (c) Heterozygosity for *P. alba* and *P. alba* var. *pyramidalis*. (d) Kinship between each sample of *P. alba* var. *pyramidalis*, *P. alba* and *P. trichocarpa*.

Leaves from 11 *P. alba* var. *pyramidalis* and 28 *P. alba* individuals were collected in Xinjiang, China in 2019 (Figure 1a. Table S1), then dried and stored in silica gel. All *P. alba* individuals were collected from natural populations. For *P. alba* var. *pyramidalis*, to reduce the effect of somatic genetic drift, we selected trees with similar diameter at breast height (~30 cm) and height (~20 m) and collected the leaves from the tip of the lowest branch for each tree. Three of the individuals sampled were been planted at Xinjiang Agricultural University in 1952, corresponding to the time when *P. alba* var. *pyramidalis* started to be widely cultivated across China (Xu, 1988). Total genomic DNA was extracted from each sample and used to construct paired‐end sequencing libraries with an insert size of 500 bp, according to the manufacturer's instructions. Then, 150 bp paired reads were generated on an Illumina HiSeq 2500. For somatic mutation validation, we randomly selected five *P. alba* var. *pyramidalis* samples to perform independent DNA extraction and sequencing in triplicate. We also accessed previously published genome resequencing data of eight other poplar species (5 *P. davidiana*, 29 *P. tremula*, 2 *P. mexicana*, 1 *P. laurifolia*, 5 *P. nigra*, 5 *P. pruinose*, 4 *P. euphratica* and 20 *P. trichocarpa*; Table S2) from NCBI. For the obtained raw reads, quality control was performed using the software *scythe* (https://github.com/vsbuffalo/scythe) and *sickle* (https://github.com/najoshi/sickle) to remove the adapter sequences and low‐quality reads. Reads that passed quality control were aligned to the *P. alba* var. *pyramidalis* reference genome (Ma et al., 2019) using BWA‐0.7.12 (Li & Durbin, 2009). The *Picard* (Broad Institute, 2019) package was subsequently used to mark duplicate reads, followed by a local realignment of reads to enhance alignments in regions around putative indels using the *Genome Analysis Toolkit* (GATK) v3.8 (Poplin et al., 2017). To exclude misalignments and ensure high‐quality variant discovery, only reads that mapped uniquely to a single place in the genome were retained for downstream analyses.

### 2.2 Variant calling and relatedness analysis

The *HaplotypeCaller* and *GenotypeGVCFs* algorithms included in GATK (McKenna et al., 2010; Van der Auwera et al., 2013) were used for multi‐sample variant calling. The obtained raw SNPs were filtered by GATK under the parameters “FS > 60 || MQ < 40 || MQRankSum < −12.5 || QD < 2 || ReadPosRankSum < −8 || SOR > 3,” and small indels were filtered using the parameters “QD < 2.0 || FS > 200.0 || ReadPosRankSum < ‐20.0” (Yang et al., 2018). The output was further filtered to remove SNPs according to the following criteria: (i) SNPs with extremely high (> threefold average depth) or extremely low (< one‐third average depth) coverage; (ii) SNPs occurring at or within 5 bp from any indels; (iii) genotypes with quality scores (GQ) < 10 and (iv) SNPs with more than 25% genotype missing rates across all samples. Subsequently, we used the *KING* algorithm (Manichaikul et al., 2010) implemented in software *VCFtools* (Danecek et al., 2011) to examine the degree of genetic relatedness between all individuals using the high‐quality SNP data. Principal component analysis (PCA) and Tracy–Widom test were performed using the software *EIGENSOFT* (Patterson et al., 2006). *PHYLIP* (Felsenstein, 2005) was used to construct the phylogenetic tree using Neighbor‐Joining method, with *P. mexicana* as the outgroup.

### 2.3 Identification of somatic mutations in *P. alba* var. *pyramidalis*

To accurately detect somatic mutations, we calculated counts of covered reads (i.e., the reads that potentially contain somatic mutations) for both forward and reverse strands, instead of only using the depth for each site. We counted the covered reads in the forward and reverse strands for all SNP candidates in each sample using the software *VarScan* (Koboldt et al., 2012). Only SNPs with both strands and a bias ratio between them less than 2 were treated as high‐quality SNPs and retained. To further minimize the rate of false discovery, we also removed the SNPs located within structural variants (SV), including inversions, translocations, deletions and inversions, which were identified using *CNVnator* (Abyzov et al., 2011), *Pindel* (Ye et al., 2009) and *Breakdancer* (Chen et al., 2009). We only considered the SVs that occurred in more than half of the samples. For indels, we used the *HaplotypeCaller* algorithm in GATK to quantify mapped read counts, and sites with less than 5 read counts and more than 10% missing rates across samples were removed.

Because some SNPs were shared by multiple individuals, we use a phasing‐separation method to identify whether those mutations were in the same haplotype. We performed phasing using a read‐based method implemented in *WhatsHap* (Martin et al., 2016) for one PacBio‐sequenced individual (Ma et al., 2019) and other Illumina‐sequenced individuals. Haplotype assembly for the PacBio sequenced individual covered 79.7% (325M/408M) of the genome with an N50 of 680 kb. Then, we mapped the phased results for all Illumina‐sequenced individuals to the haplotype assemblies of the PacBio individual using a custom Perl pipeline.

To identify derived mutations, we used *P. alba* and *P. tremula* as outgroups. The sites that exhibited the same homozygous genotype in *P. alba* and *P. tremula* were used as the ancestral state for these identified somatic mutations. The derived mutations of *P. alba* var. *pyramidalis* were used for phylogenetic network analysis using *SplitsTree* (Huson & Bryant, 2006).

Amino acid substitutions and their effects on protein function were predicted using *SIFT* (Ng & Henikoff, 2003), *PROVEAN* (Choi et al., 2012) and *PolyPhen2* (Adzhubei et al., 2010) with default parameters. Nonsynonymous mutations with *SIFT* scores ≤0.05, *PROVEAN* scores < −2.5 and *PolyPhen* prediction scores >0.5 were treated as putative deleterious mutations.

### 2.4 Estimation of the false‐positive rate and false‐negative rate

To determine the false‐positive rate of our variant‐calling pipeline, we randomly selected five *P. alba* var. *pyramidalis* samples and sequenced each sample three times independently, generating 24.7× sequencing data each time, on average. The same pipeline was then used to detect somatic mutations, which were compared to mutations detected in the other replicates. The variant sites identified in all three replicates were considered as true mutations, while other sites were considered as erroneously called mutations. Among the 1898 candidate mutations identified in these five samples, we found that only 44 (3.2%) of them were erroneously called mutations, indicating a low false‐positive rate. In addition, we estimated the false‐negative rate using a reads‐modified method (Keightley et al., 2015; Xie et al., 2016). We randomly simulated 1000 single‐point substitution mutations across the whole genome according to the approach described in Keightley et al. (2015). Since poplars are diploid, these substitutions represent homozygous derived sites. We calculated the count of covered reads for both forward and reverse strands that contained somatic mutations. Mutations were treated as callable sites only if the number of covered reads was greater than 3. Out of these 1000 synthetic mutations, 60.4% (604) were callable. Of all the callable synthetic mutations, 0.8% (8) were filtered through our pipeline. Therefore, we estimated that the false‐negative rate was 59.6%, slightly lower than the rates estimated in wild peach (62.8%) (Hofmeister et al., 2020) and oak (69.4%) (Plomion et al., 2018). The high false‐negative rate might be caused by high rates of heterozygosity or low sequencing depth.

### 2.5 Statistical analysis

Mutation rate was estimated according to the following formula:

$$\text{Mutation rate}\;\left(\mathrm{per}\;\text{base}\;\mathrm{per}\;\text{year}\right)=\frac{\text{Mean mutation count}\;\mathrm{per}\;\text{sample}}{\left(2019-1952\right)*2*408M}*\frac{1-\text{false negative rate}}{1-\text{false positive rate}}$$

2019 and 1952 represent the collection time and the planting time, respectively. 408M represents the genome size. Since *P. alba* var. *pyramidalis* is diploid, the total DNA base count is 2*408M.

The trinucleotide context of point mutations was counted with the mutation at the first, second and third positions of the triplet, and the mutation rate per given trinucleotide triplet was calculated accordingly. The dinucleotide context was counted from the first and second nucleotide position of each sequence. Mutation virtualization was performed using *sigfit* (Gori & Baez‐Ortega, 2018).

To estimate the recombination rate of the sexually reproducing ancestors of *P. alba* var. *pyramidalis*, we calculated the population recombination rate of *P. alba*. First, we used *Beagle* (Browning et al., 2018) to phase all SNP sites across the *P. alba* individuals and then calculated the population recombination rate with *FastEPRR* (Gao et al., 2016) using 50 kb nonoverlapping windows. We classified the windows into seven groups according to each window's count of somatic SNPs. Lastly, the mean population recombination rate of each category was calculated, and regression analysis was performed on the resulting data using the R package *stats* (R Core Development Team, 2019). Enrichment analysis was performed using *TopGO* (Alexa & Rahnenfuhrer, 2010) and *REVIGO* (Supek et al., 2011).

## 3 RESULTS

### 3.1 Plant sample and population structure

Our dataset combines newly generated whole‐genome resequencing data (11 *P. alba* var. *pyramidalis* and 28 *P. alba* individuals) with publicly available resequencing data from 71 individuals representing another eight poplar species (Table S2).

After mapping reads to the *P. alba* var. *pyramidalis* reference genome, the average sequence depth of individuals that were sequenced three times was approximately 87.1×, with other samples at approximately 21.0× (minimum 17.1×; Table S1). In total, 3.3 M SNPs and 0.25 M indels were identified for *P. alba* var. *pyramidalis*, and 7.93 M SNPs and 2.27 M indels were identified for *P. alba*.

With the aid of high‐quality variants from six other *Populus* species distributed across Xinjiang (Table S2) and the *P. mexicana* outgroup, we built a comprehensive phylogenetic tree. The tree confirmed that *P. alba* var. *pyramidalis* has the closest relationship with *P. alba* (Figure 1b), which is consistent with previous studies (Xu, 1988; Yin, 2006). To investigate the population structure and relationships within *P. alba* var. *pyramidalis*, we performed PCA and KING‐ship analysis based on the sequencing data. We found that all *P. alba* var. *pyramidalis* individuals were tightly grouped together (Figure S1) and exhibited “duplicate” relationships (Figure 1d). This coincides with the historical record that *P. alba* var. *pyramidalis* was domesticated and clonally propagated by means of branch cuttings from one or a very limited number of *P. alba* individuals (Xu, 1988). Our results also suggested that the domesticated *P. alba* var. *pyramidalis* individuals had significantly higher levels of heterozygosity than other *P. alba* individuals (Figure 1c).

### 3.2 Somatic mutation and mutation rate

After strict filtering, a total of 3037 somatic SNPs and 637 somatic indels were retained in the 11 *P. alba* var. *pyramidalis* individuals (Figure S2). Most of the somatic mutations were in intergenic and intronic regions, and significantly fewer mutations were located in coding regions (χ_1_^2^ with Yates correction = 7.50, *p* = 0.006) or exonic regions (χ_1_^2^ with Yates correction = 5.6, *p* = 0.018). Furthermore, most mutations were sample‐specific, and only 250 mutations (208 SNPs and 42 indels) were shared by more than two samples (Figure S2a,b). For each sample, somatic mutations were distributed randomly throughout the genome (Figure S3).

**FIGURE 2:**
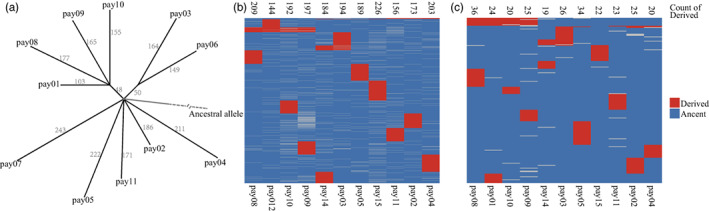
Derived mutation network tree (a) and spectrum (b, c). (a) Derived mutation network tree based on the SNP dataset. Spectrum for SNPs (b) and indels (c). The ancestral state was identified using *P. alba* and *P. tremula* as outgroup.

A total of 1851 SNPs and 230 indels were identified as derived mutations in *P. alba* var. *pyramidalis* (Figure 2b,c). Similar to the shared somatic mutations, most were sample‐specific, with only 135 (113 SNPs and 22 indels) shared by two or more samples. Based on the derived mutation spectrum, we obtained a clear propagation pedigree of *P. alba* var. *pyramidalis* (Figure 2). The result suggested that pay01, pay08, pay09 and pay10 shared the most somatic mutations and were clonally propagated from a common plant. A few mutations were also shared between other individuals (e.g., between pay03 and pay06, and among pay11, pay02 and pay04; Figure 2).

Based on the propagation pedigree of *P. alba* var. *pyramidalis* (Figure 2), we estimated that each individual had 312.5 somatic SNPs and 59.9 somatic indels on average (Figure S2, Table S3). Given that three *P. alba* var. *pyramidalis* individuals were clonally propagated in 1952 at Xinjiang Agricultural University, and that since then *P. alba* var. *pyramidalis* has been widely cultivated across China, we speculated that the derived somatic mutations in our samples accumulated during this time (i.e., 1952–2019, or 67 years). Combining with the false‐positive (3.2%) and false‐negative rate (60.4%), the somatic point (SNP) mutation rate was estimated to be 9.24 × 10^−9^ per bp per year (95% CI: 8.39 to 1.01 × 10^−9^, Table S3). For indels, the mutation rate was estimated as 1.77 × 10^−9^ per year per site (95% CI: 1.55 to 1.99 × 10^−9^, Table S3).

To test whether the shared somatic SNPs were in same haplotype, we phased the somatic SNPs. For all somatic SNPs, 86.0% (2612) were phased with nearby SNPs, while 60.8% (1845) sites were phased in all *P. alba* var. *pyramidalis* samples. As for the shared SNPs, 81.7% (170) were phased with nearby SNPs, of which 66.8% (141) were phased in all samples. For those phased sites, we found that all shared somatic mutation were in the same haplotype, which indicated the shared mutation was generated by the parent plant before cutting. This further demonstrates the high quality of SNP calls in our result.

### 3.3 Mutation is AT‐biased especially in asexual reproduction

**FIGURE 3:**
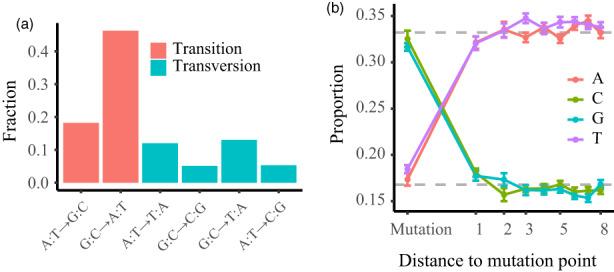
Somatic mutation tendentiousness. (a) Spectrum of somatic mutations. (b) Proportion of base content for distance from somatic mutations. The gray dashed line represents genome‐wide averages for A/T (upper) and C/G (lower).

We found that the types of premutated bases at mutated sites were significantly different from those expected by chance at the whole‐genome level and within the regions adjacent to mutations. Contrary to the pattern commonly observed at the genomic level, our results indicated a higher GC content than AT content for premutated bases (Figure 3a), suggesting that somatic mutations tend to occur in regions with high GC content. We consistently found a higher proportion of transitions or transversions from GC to other bases than from AT to other bases (Figure 3a). The primary mutation type was G/C‐to‐A/T transition (46.3%), while G/C‐to‐C/G transversion accounted for the lowest proportion of mutations (5.1%, Figure 3a). After correcting for nucleotide content, AT‐biased mutations (G/C‐ > A/T per G/C) occurred at a 5.11‐fold higher rate than mutations in the opposite direction (A/T‐ > G/C per A/T) in *P. alba* var. *pyramidalis*.

We also noticed that mutation tends to occur in premutated C/G bases (Figure 3b) and that the highest proportion of mutations of dinucleotides (per class of site) was from CpG sites after correcting for genomic background (Table S4). For the proportion of triplets of the somatic mutation, we found that CHG sites were significantly enriched in mutations but CHH sites were not (Figure S4).

### 3.4 Mutations tend to occur in heterozygous regions and are unrelated to recombination rate

**FIGURE 4:**
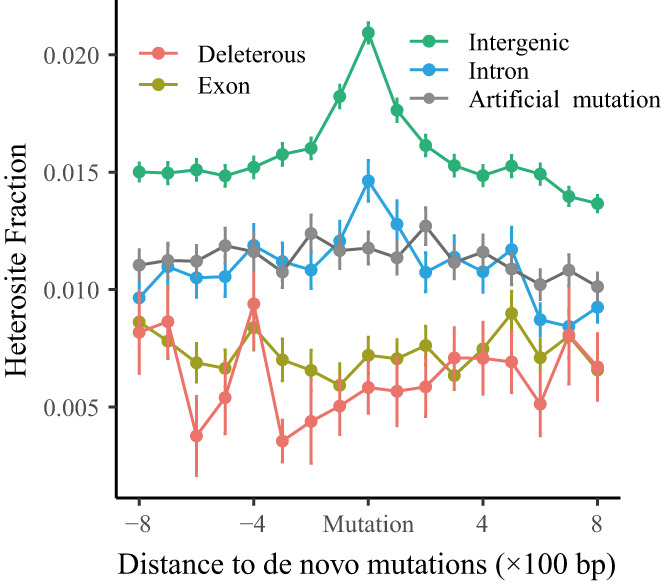
Relationship between nucleotide diversity and the distance to somatic mutations for intergenic, intron, exon, deleterious and artificial mutations.

Mutations from homozygous to heterozygous states (*n* = 2328) accounted for the largest proportion of the 3037 somatic mutations (76.7%; Table S5). These formed an even greater proportion of the shared mutations (*n* = 172, 82.7%; Table S5). We found no significant relationship between mutation hotspots and ancestral recombination rate (*p* = 0.22, Figure S5). After performing heterozygous analysis, we found that the somatic mutations tended to occur in high‐heterozygous regions (Figure 4), consistent with previous work (Yang et al., 2015). This trend was evident in the intergenic and intronic regions, but not in the exon regions (Figure 4). To verify the heterozygous‐mutation tendency, we randomly selected 1000 sites in the whole genome and calculated the fraction of heterozygous sites in its 1.2 kb flanking regions, and no homozygous biases were observed even after stringent filtering (Figure S6).

### 3.5 Deleterious mutations for somatic and derived variants

The SNP annotation algorithms of *SIFT*, *PROVEAN* and *PolyPhen2* resulted in 68, 68 and 78 deleterious somatic mutations, respectively (Figure S7). Among them, 44 were supported by all the three algorithms, which accounted for 1.45% of all somatic mutations (Table S6). Interestingly, we found that all deleterious mutations were sample‐specific and maintained at a heterozygous state, with the counts ranging from 1 in pay10 to 6 in pay02, pay05, pay07 and pay09 (Table S6).

To further examine the mutational burden associated with rapid clonal domestication, we used *P. alba* and *P. tremula* as outgroups to identify the derived alleles in *P. alba* var. *pyramidalis*, removing sites with a minor allele frequency (MAF) <0.1. 556,000 derived alleles were identified, most of which were heterozygous (94.8%) and very few of which were fixed (4.1%). Only 695 (0.12%) derived alleles were annotated as deleterious mutations, all of which were heterozygous. The functions of the genes containing derived deleterious mutations were varied (Figure S8), and only one GO term regarding membrane transport was enriched (*p* < 10^−5^, false discovery rate [FDR] = 6 × 10^−3^).

**FIGURE 5:**
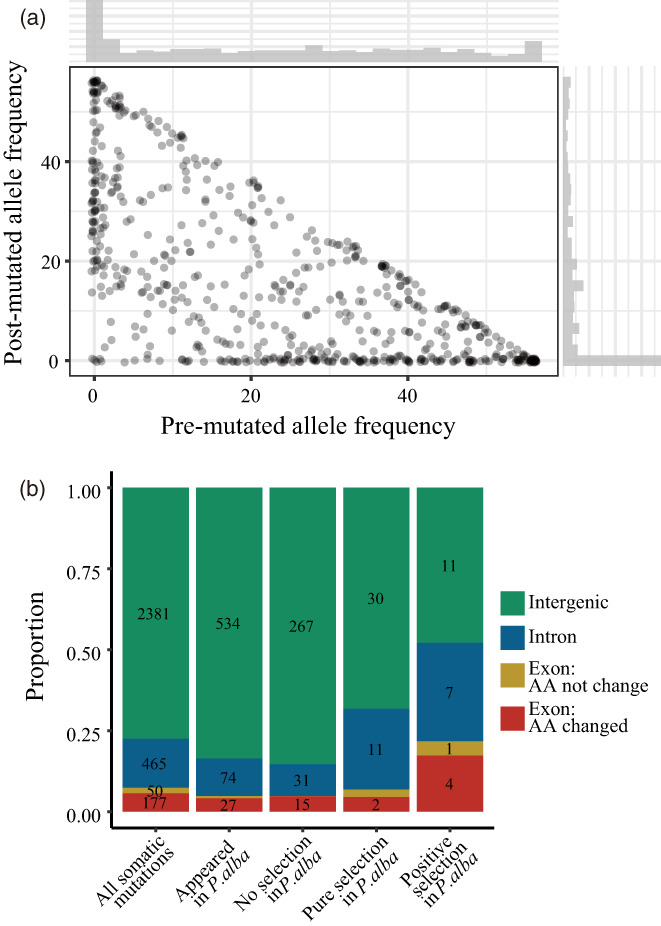
Selection of somatic alleles in *P. alba*. (a) For each somatic mutation in *P. alba* var. *pyramidalis*, corresponding premutation and postmutation allele frequency in *P. alba*. (b) Distribution of SNPs under selection in *P. alba*.

In addition, we also found that somatic mutation of *P. alba* var. *pyramidalis* may be under selection in *P. alba*. We calculated the allele frequencies of sites pre‐ and postmutation in *P. alba*. Then, we treated somatic mutations with >95% postmutation allele frequency as positive selection and > 95% premutation allele frequency as purifying selection (Figure 5a). We found that mutations under positive or purifying selection were more highly enriched in introns. The proportion of positively selected mutations occurring in exons or changing amino acids was much higher than purifying selection and nonselection mutations. (Figure 5).

## 4 DISCUSSION

### 4.1 High heterozygosity and environmental factors may be the key to high somatic mutation rate

The somatic mutation rate of *P. alba* var. *pyramidalis* was estimated at 9.24 × 10^−9^ per bp per year for SNPs and is similar to individual rates (Table S3). This rate is much higher than the rate estimated for *P. trichocarpa* (1.99 × 10^−9^) (Hofmeister et al., 2020) and most related species (e.g., peach, oak and *Arabidopsis*, Table 1). The somatic mutation rate for indels is 1.77× 10^−9^ per bp per year. The ratio of somatic mutation rates between SNPs and indels is 5.21, which is very close to 5.13, the ratio in peach (Xie et al., 2016). Our results showed that heterozygosity may be intimately linked to increased mutation rates (4.64‐fold higher mutation rate and 1.61‐fold higher level of heterozygosity in *P. alba* var. *pyramidalis* compared to *P. trichocarpa*) (Figure 1c). This relationship is also observed in sexually reproducing species, such as rice (Yang et al., 2015) and *Arabidopsis* (Yang et al., 2015), as well as in asexually reproducing species such as peach (Xie et al., 2016; Table 1). Furthermore, the observation of higher mutation rates in the vicinity of heterozygous sites (Figure 4) is consistent with previous findings that mutations in these regions occur more frequently than expected by chance (Xie et al., 2016; Yang et al., 2015). However, it is notable that enhanced mutation rates in *P. alba* var. *pyramidalis* (4.64) are much higher than those in peach (1.67), *Arabidopsis* (3.72) and rice (3.40) (Table 1), suggesting that there may be other factors responsible for the elevated mutation rate. Previous studies have shown that environmental factors such as tissue culture, shock and stress can cause the mutation rate to increase (Gill et al., 1995; Jiang et al., 2014). Thus, any changes to the environment introduced during cutting propagation and growth (e.g., changing rooting solution or nutrient solutions or applying fungicide [Agricultural Science Institute of the fourth Agricultural Division, 1962; Haide, 1981]) that directly influence the balance between cytokinin and auxin levels may have an impact on the mutation rate and contribute to accumulation over time (Prusinkiewicz et al., 2009; Schaller et al., 2015). Moreover, all samples of *P. alba* var. *pyramidalis* were collected from locations that are cloudless and exposed to naturally high levels of ultraviolet light. The strong ultraviolet light exposure could also lead to an elevation of mutation rate (Lindberg et al., 2019).

**TABLE 1:** Mutation rates found in previous studies.

Species	Mutation type	Heterozygous	Plant life form	Mutation rate (×10^−9^)^a^	DOI
*Arabidopsis thaliana*	Somatic	No	Annual herb	4.35	10.1371/journal.pbio.3000191
*Arabidopsis thaliana*	Sexual	No	Annual herb	7.20	10.1038/nature14649
*Arabidopsis thaliana*	Sexual	Yes	Annual herb	26.80	10.1038/nature14649
*Oryza sativa*	Somatic	No	Annual herb	9.01	10.1371/journal.pbio.3000191
*Oryza sativa*	Sexual	No	Annual herb	3.20	10.1038/nature14649
*Oryza sativa*	Sexual	Yes	Annual herb	11.00	10.1038/nature14649
*Prunus mira*	Somatic	No	Perennial tree	0.13^b^	10.1371/journal.pbio.3000191
*Prunus persica*	Somatic	No	Perennial tree	0.82^b^	10.1371/journal.pbio.3000191
*Prunus mume*	Somatic	No	Perennial tree	2.17	10.1371/journal.pbio.3000191
*Prunus mira and P. persica*	Sexual	No	Perennial tree	8.262^c^	10.1038/nature14649
*Prunus davidiana x P. persica*	Sexual	Yes	Perennial tree	13.80	10.1098/rspb.2016.1016
*Brachypodium distachyon*	Somatic	No	Annual herb	6.13	10.1371/journal.pbio.3000191
*Fragaria vesca*	Somatic	No	Perennial herb	6.37	10.1371/journal.pbio.3000191
*Salix suchowensis*	Somatic	No	Perennial shrub	2.58	10.1371/journal.pbio.3000191
*Quercus robur*	Somatic	No	Perennial tree	0.20	10.1101/149203
*Eucalyptus melliodora*	Somatic	No	Perennial tree	2.75	10.1101/727982
*Populus Trichocarpa*	Sexual	No	Perennial tree	1.99	10.1186/s13059‐020‐02162‐5
*Populus alba* var. *pyramidalis*	Somatic	Yes	Perennial tree	9.24	This study

^a^ Mutation rate per bp per year corrected for accessible genome regions.

^b^ Mean mutation rate of all samples.

^c^ Yang peach selfed mutation rate is 7.77 and for 200‐year‐old is 9.48.

### 4.2 Relationships between somatic mutation, GC content and recombination

Our results indicate that somatic mutations tend to occur in regions with high GC content. In addition, we found a higher proportion of mutations from G/C to other bases than other transitions or transversions (Figure 3). Previous studies in mouse (Clément & Arndt, 2013), yeast (Kiktev et al., 2018) and many prokaryotic species (Shee et al., 2012; Weissman et al., 2019) have also revealed the genomic regions with high GC content tend to be somatic mutation hotpots. These regions are more likely to be the sites of DNA methylation, which can influence DNA repair and in turn lead to altered mutation rates (Dubrovina & Kiselev, 2016). In this study, we found the mutation rate in the “CpG” category was significantly higher than that of other nucleotide‐context categories, while the mutation rate in the “CHG” category was only slightly higher than that of other categories (Figure S4). We suggest that somatic mutations concentrated around CpG sites in *P. alba* var. *pyramidalis* might be caused by DNA methylation changes, consistent with previous work in *P. trichocarpa* that found higher somatic epimutation rates in mCG than in mCHG in this species (Hofmeister et al., 2020),

We also found a higher rate of transitions in somatic mutations. The transition/transversion (T_i_/T_v_) ratio in this study (1.64) was similar to that found in a recent study (Soorni et al., 2017) where the average Ts/Tv ratio for 69 cannabis individuals was 1.65. Additionally, this ratio is similar to oil palm (1.67), though significantly higher than maize (1.02) (Batley et al., 2003; Pootakham et al., 2015).

A previous study in sexually reproducing bumblebees detected a weak signal of recombination‐associated mutations, and both the recombination and mutation had positive correlations with GC content (Liu et al., 2017). Here, however, we did not observe such a correlation between somatic mutation and the recombination rates of the sexually reproducing ancestor *P. alba* (Figure S5). These conflicting findings have several potential explanations, including (1) the correlation is very weak even under sexually reproducing systems, (2) we tested the relationship with the recombination rate from *P. alba*, not that of *P. alba* var. *pyramidalis*, (3) these patterns may differ between insects and plants, or they may be species‐specific, and (4) we focus only on somatic mutation and use different methods.

### 4.3 Short but strong domestication effect was observed

Artificial selection is a powerful evolutionary force and can mask recessive deleterious mutations by maintaining them in heterozygous states during domestication as recent work showed in cassava (Ramu et al., 2017). Consistent with this study, we also found that all deleterious somatic mutations detected in *P. alba* var. *pyramidalis* appeared in the heterozygous state, and none of these deleterious mutations were shared by two or more individuals, even among individuals cloned from a common plant. This may be the result of the species' short domestication history accompanied by artificial selection associated with cutting propagation. A heterozygous genetic background may provide an evolutionary advantage, as it can buffer the effects of deleterious genetic mutations and increase a plant's survival rate under stress (Ramu et al., 2017). Recombination can effectively eliminate deleterious mutations from the genome (Keller & Knop, 2009). For sexually reproducing species, such as human (Hussin et al., 2015), maize (Rodgers‐Melnick et al., 2015) and fruit fly (Haddrill et al., 2007), deleterious mutations tend to be concentrated in low‐recombination regions. For asexual species like *P. alba* var. *pyramidalis*, however, there is limited potential for recombination‐mediated removal of deleterious mutations. As a result, all mutations are transmitted to the offspring. Although artificial selection tends to favor individuals with fewer harmful mutations because breeders select the best individuals (Ramu et al., 2017), the number of deleterious somatic mutations is on average 4.0 per individual in *P. alba* var. *pyramidalis*, which could continuously increase during clonal propagation and domestication of this species. Our results also indicated that fewer somatic mutations occurred in exonic regions and that the association between heterozygosity and mutation is weaker in exonic regions compared with intronic regions. This pattern is likely due to stronger purifying selection in transcribed exonic regions than in intronic regions, which removes more somatic mutations. On a large scale, prolonging a plant's normal lifespan through cultivation and cloning may lead to a substantial mutational load with potentially deleterious effects. Choosing ancestors with low mutational loads as measured by phenotypic and genomic screening will help reduce the accumulation of deleterious mutations during clonal propagation of this species (Horvath & Barrangou, 2010).

On the other hand, some somatic mutations may be beneficial and afford resistance to stress and/or agronomic traits as suggested by recent work in orange (Wang et al., 2021). In our study, we found that most mutations in the exons result from purifying selection, and positive selection had a significant influence on gene function (Figure 5b). These findings suggest that novel mutations could result in the emergence of advantageous phenotypes to plants. For example, we found that some somatic mutations occurred in genes that may be involved in innate immunity and plant defensive response. We found a mutation in gene *Chr7.1745*, which is homologous to gene *AT1G04510* (*MOS4‐ASSOCIATED COMPLEX 3A*, *MAC3A*) in *Arabidopsis*. The gene is associated with the MOS4‐Associated Complex (MAC), which is associated with enhanced resistance to several pathogens (Monaghan et al., 2009). In addition, we found another mutation in *Chr10.1859*, which is homologous to *AT2G36490* (*REPRESSOR OF SILENCING1*, *ROS1*) in *Arabidopsis*. *AT2G36490* is associated with several important functions, including heat stress resistance and DNA demethylation regulation (Kim, Kidokoro, et al., 2020; Kong et al., 2020; Malabarba et al., 2021). Such mutations in genes with important functions may enhance an individual's defensive capabilities and its adaptability to a changing environment.

## 5 CONCLUSIONS

The combination of population genomic data and clear propagation pedigrees allowed us to assess the incidence rate and pattern of somatic mutations in *P. alba* var. *pyramidalis*. Results reported here demonstrate that somatic mutation is heterozygous associated, AT‐biased and not related to recombination rates. The high somatic mutation rate supports the view that high heterozygosity and the presence of environmental mutagens (e.g., UV radiation) can increase the incidence of mutation. This study is the first to investigate: (1) the somatic mutation rate of pay by using genomic data and propagation pedigrees‐based method, (2) the pattern of somatic mutation in short domesticated perennial plants, and (3) the potential consequences of artificial selection acting on deleterious mutations. The results have broad implications for asexual perennials because somatic mutants are frequently observed in such plants, which are propagated using clonal breeding techniques. However, we only focused on SNPs and INDELs within a limited sample size. Future studies involving a wider range of individuals representing different clonal histories and environmental stresses combined with transcriptomic and methylation data are needed to enhance our understanding of somatic mutations in this species and to facilitate its clonal breeding program.

## CONFLICT OF INTEREST

The authors declare that they have no conflicts of interest.

## Supporting information


Figure S1‐S8

Table S1‐S6

## Data Availability

The sequencing data that support the findings of this study were deposited in the National Genomic Data Center (https://ngdc.cncb.ac.cn/) under accession PRJCA005745. Scripts in this study are available from website at https://github.com/StarSkyZheng/pay_somatic_study.
