# Supplementary material for: Somatic mutations during rapid clonal domestication of Populus alba var. pyramidalis
Source: Evol Appl. 2022 Oct 4;15(11):1875–87. doi: 10.1111/eva.13486 (PMC9679227; doi:10.1111/eva.13486)

**SUPPLEMENTARY DATA**

Somatic mutations during rapid clonal domestication of Populus alba var. pyramidalis

# Figure S1. PCA analysis of *P. alba* var. *pyramidalis* and *P. alba*.


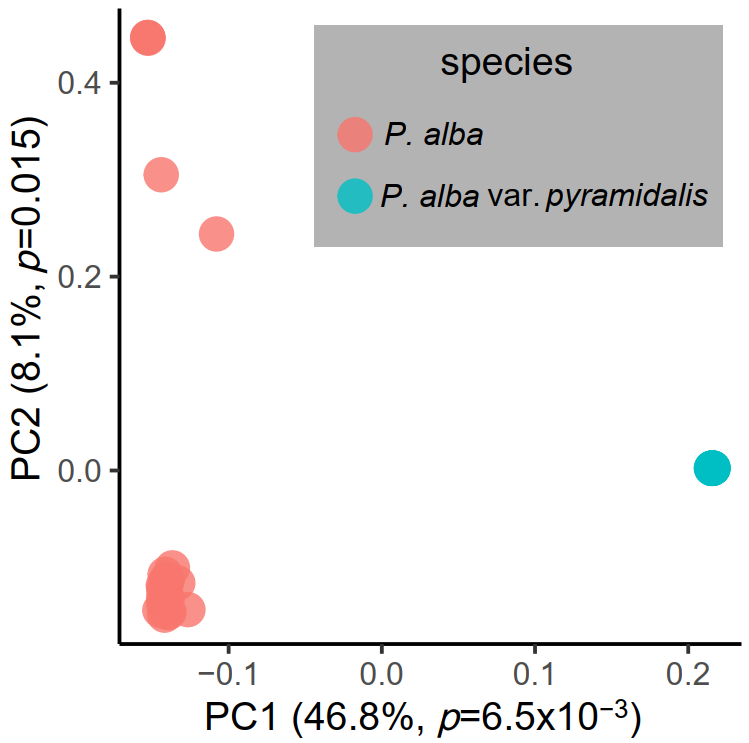


# Figure S2. Somatic mutation network tree (A, B) and spectrum (C, D) for SNPs (A, C) and InDels (B, D).


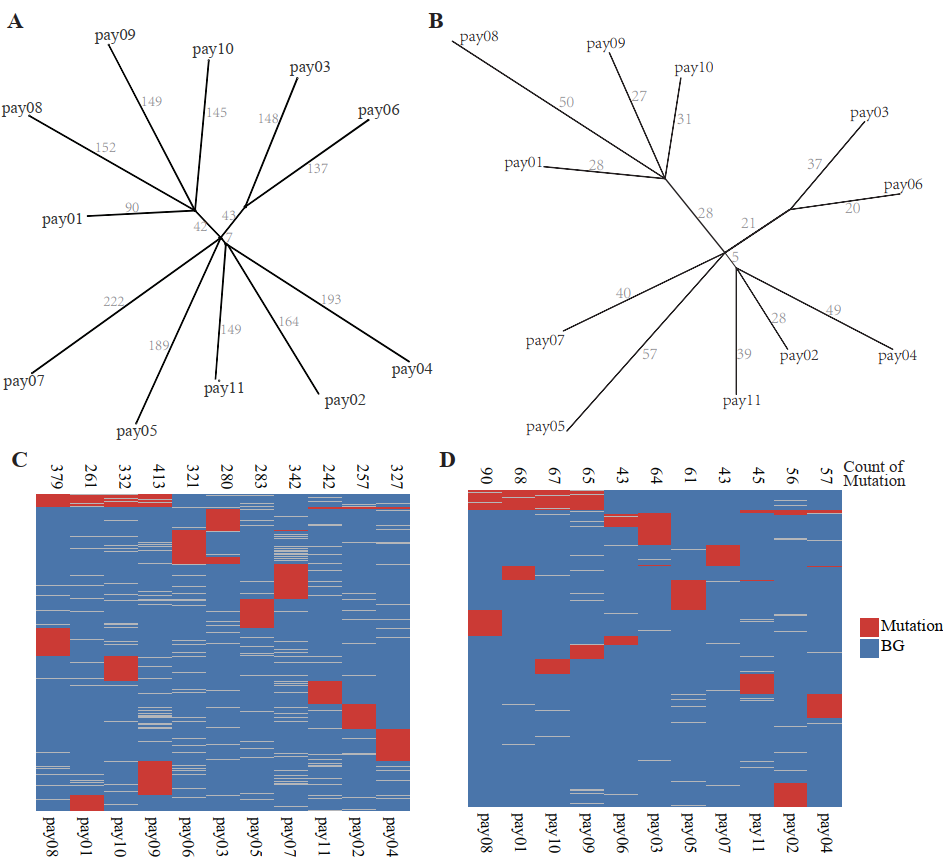


# Figure S3. Distribution of somatic SNPs in the whole genome.


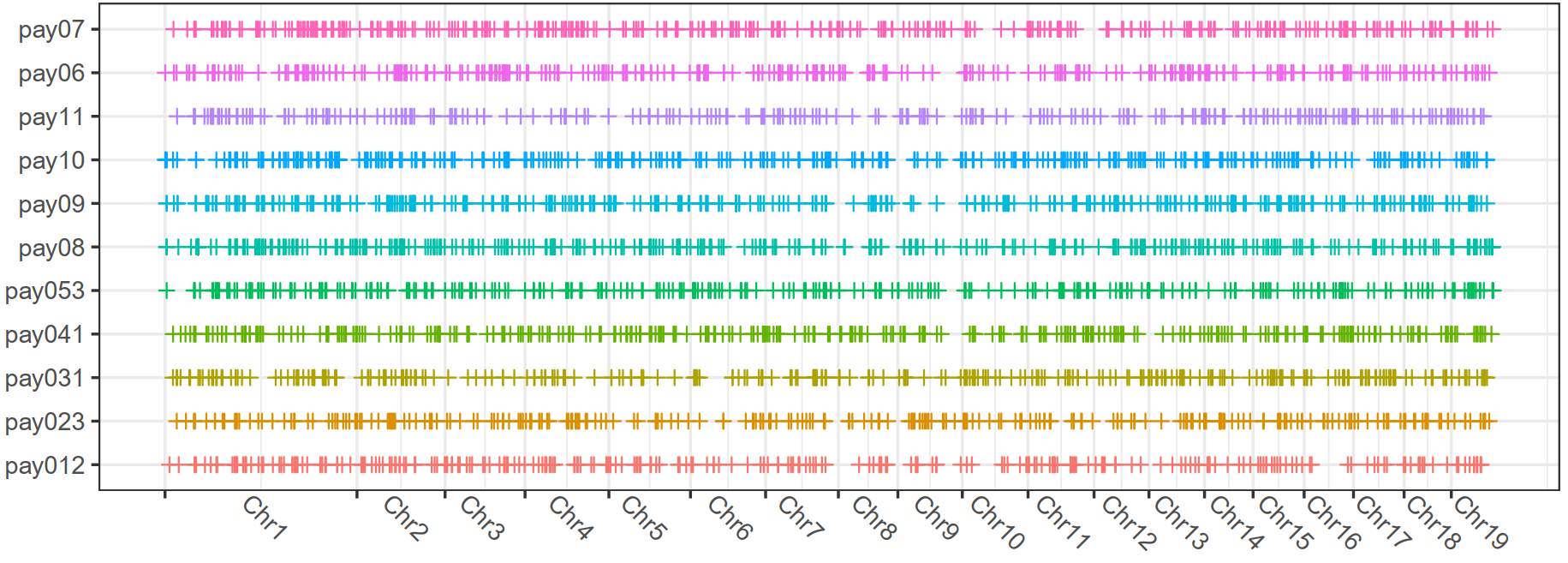


# Figure S4. Trinucleotide-context mutational spectrum of somatic SNPs which was been correcting for genomic background.


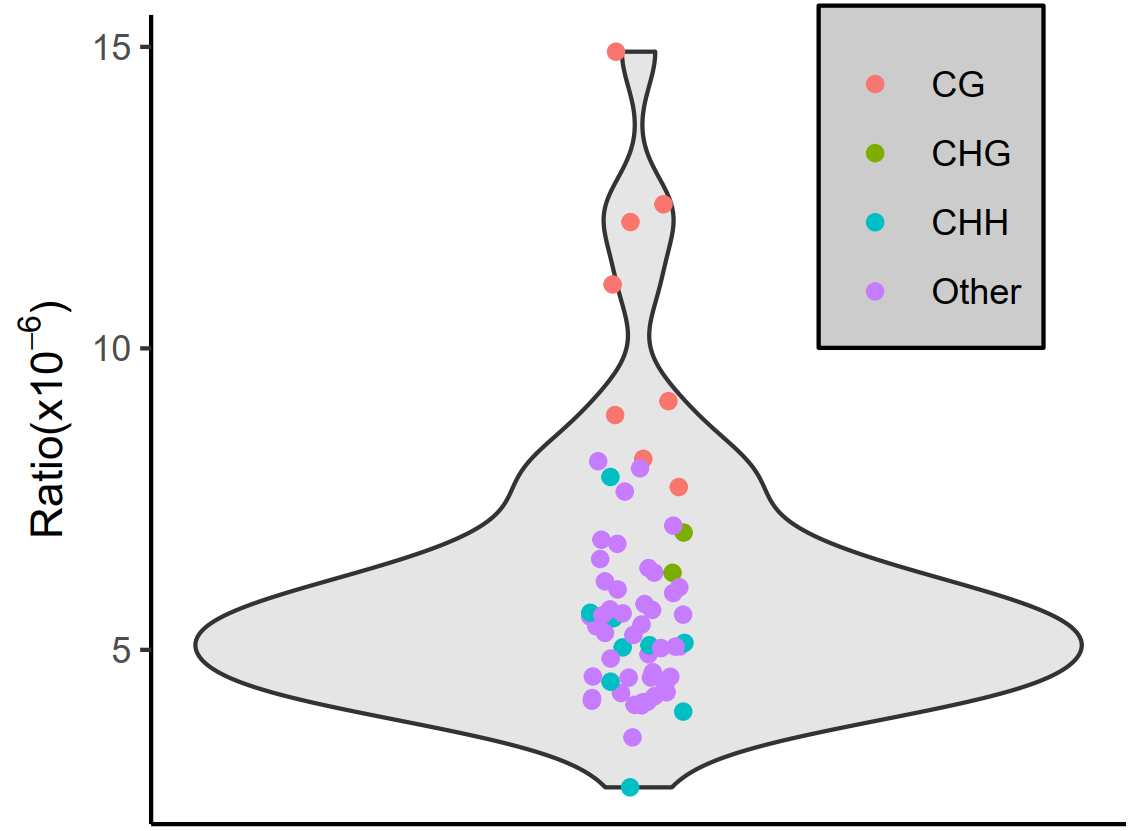


# Figure S5. Correlation ship of ancient’s (P. alba) population recombination rate and SNP count in the window.


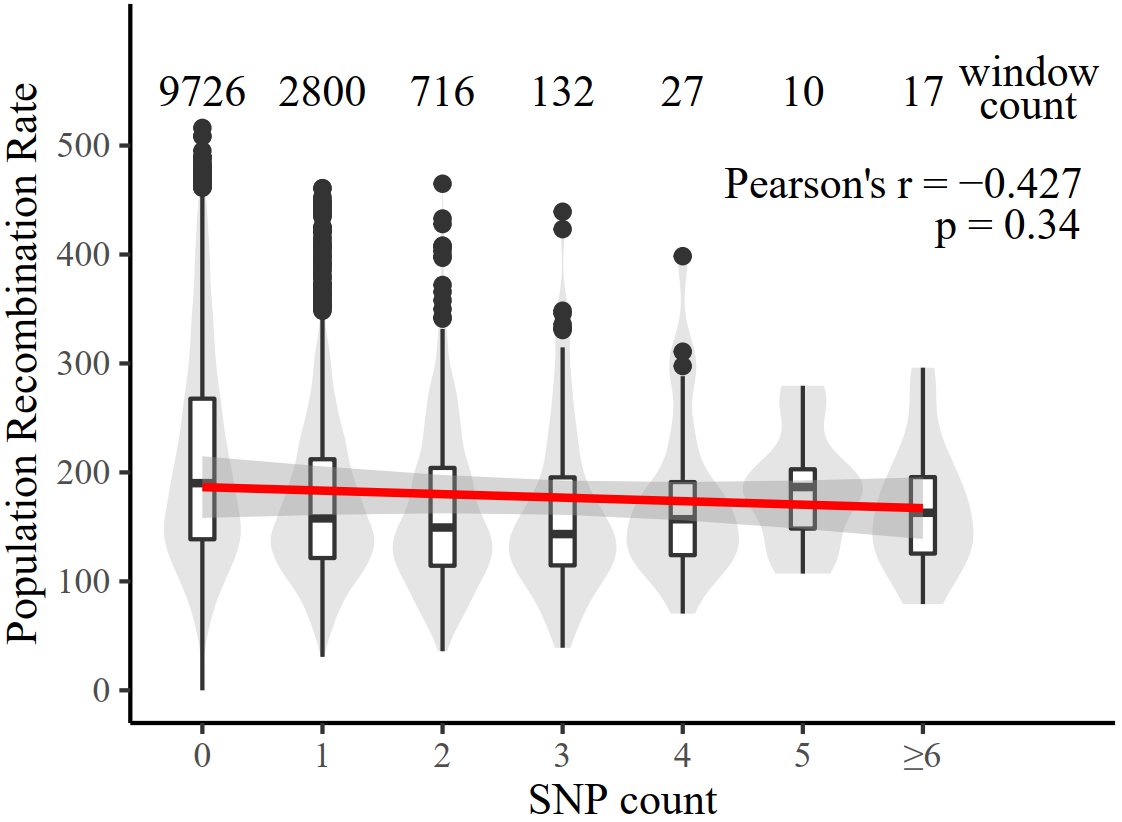


# Figure S6. Relationship between nucleotide diversity and the distance to the somatic mutations for filtered real data, simulated data and filtered simulated data.


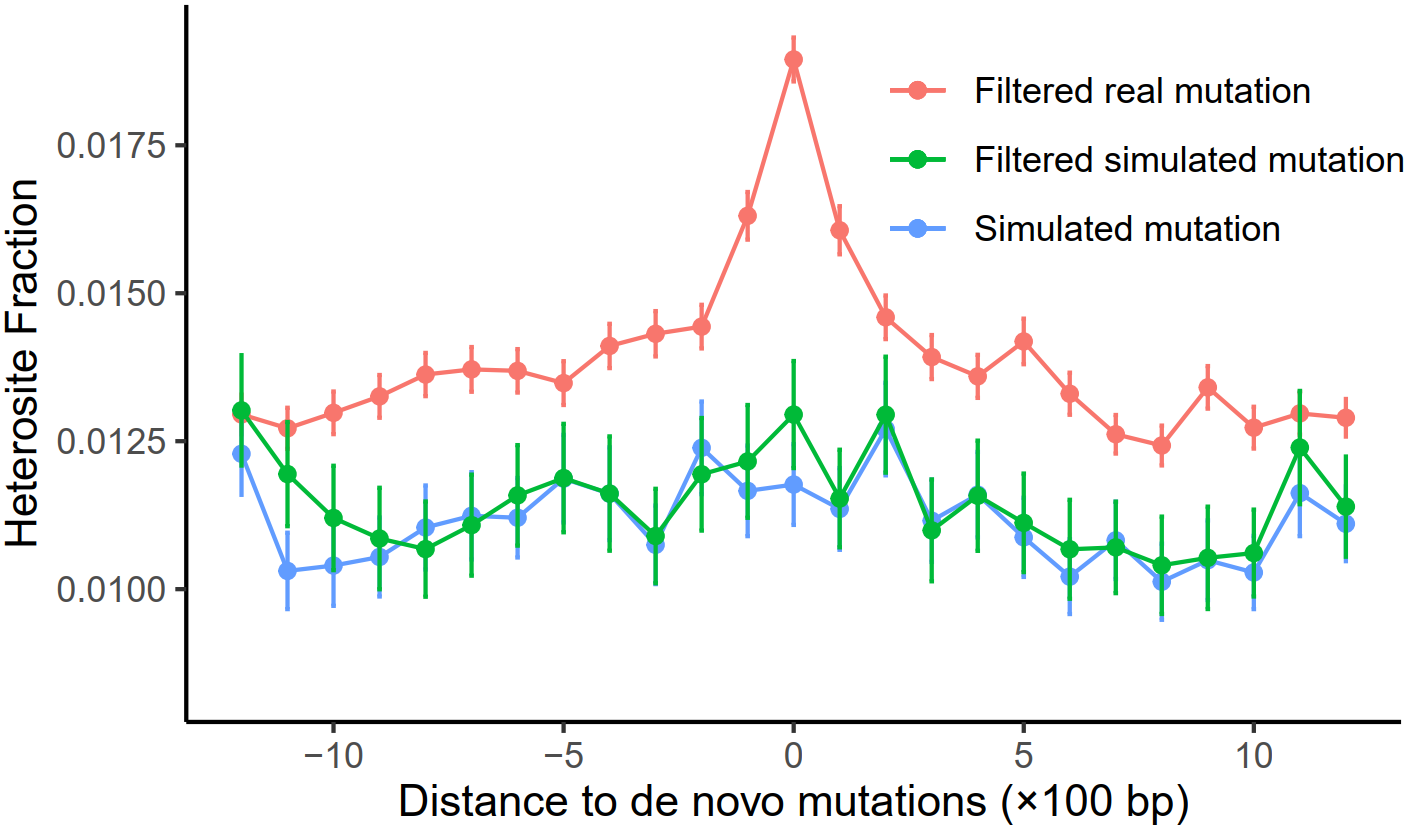


# Figure S7. Deleterious mutation annotation result for three algorithms.


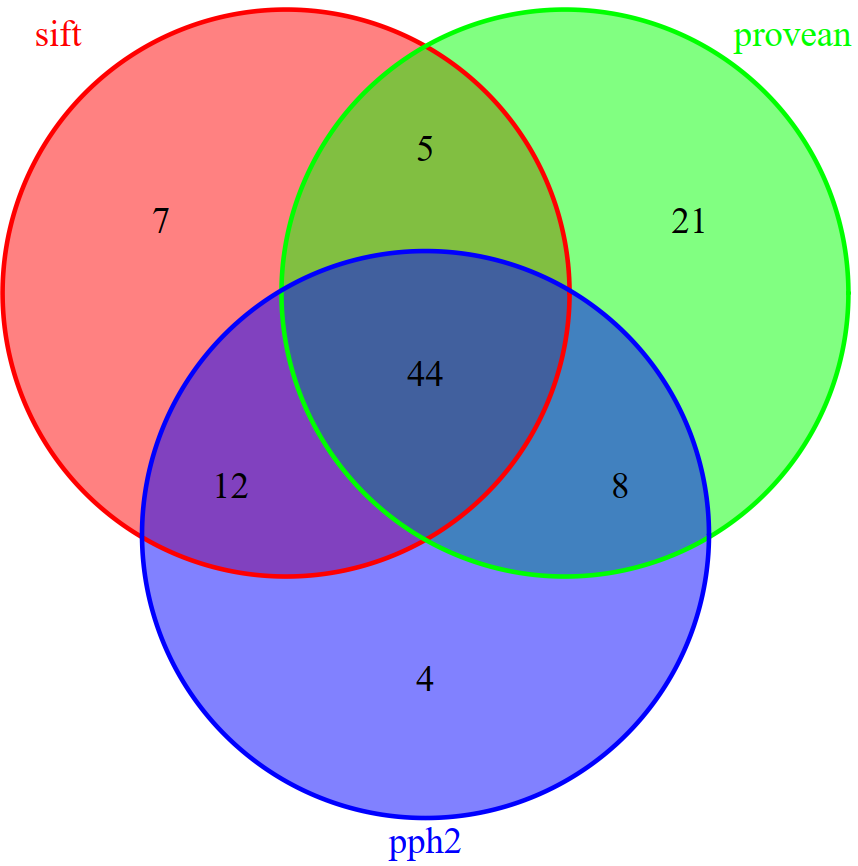


# Figure S8. Gene ontology enrichment of derived sites.


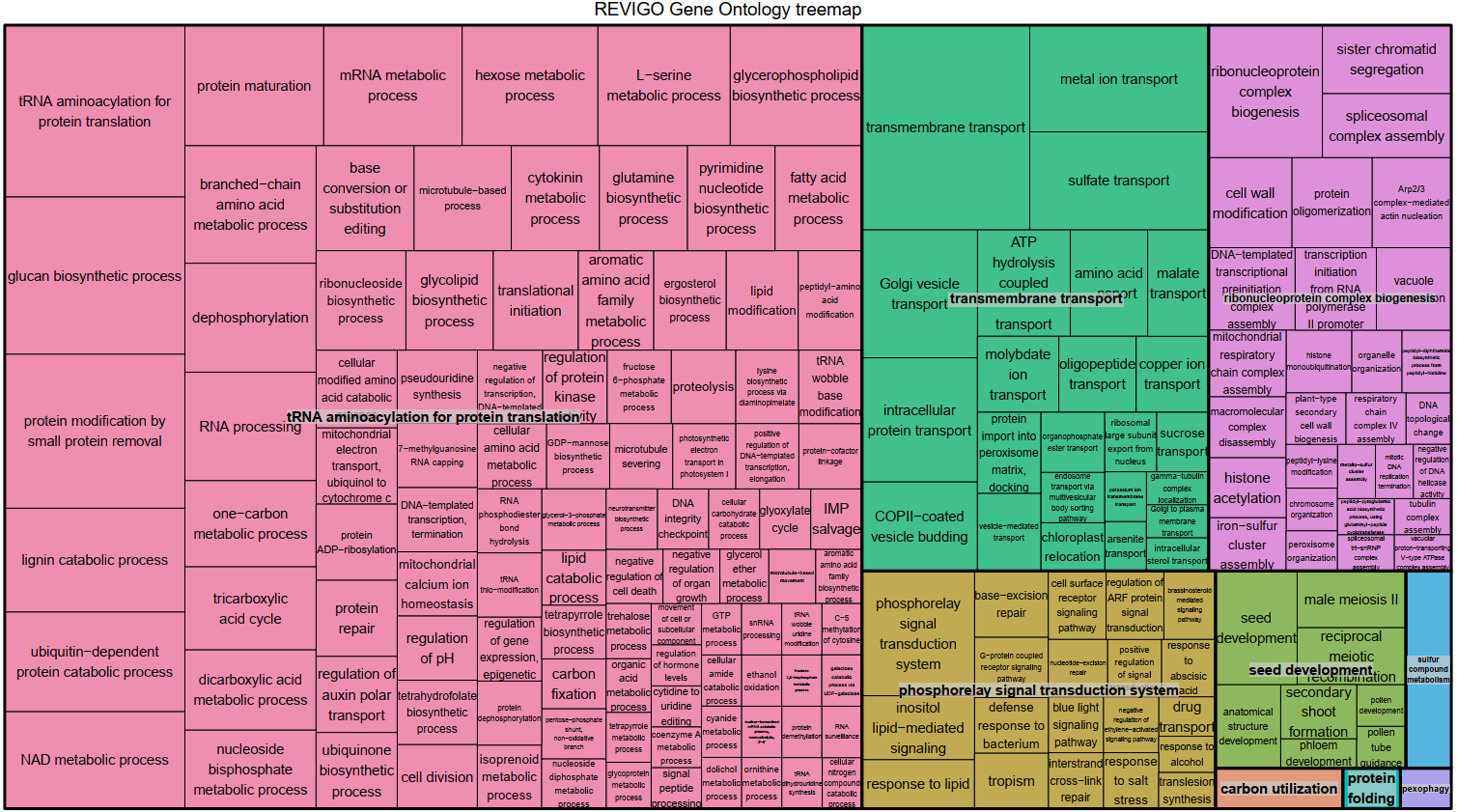

Supplement: Supplementary file 1 — Figure S1‐S8 [file EVA-15-1875-s001.docx]
